# Purple corn extract induces long-lasting reprogramming and M2 phenotypic switch of adipose tissue macrophages in obese mice

**DOI:** 10.1186/s12967-019-1972-6

**Published:** 2019-07-23

**Authors:** Federica Tomay, Alessandra Marinelli, Valerio Leoni, Claudio Caccia, Andrea Matros, Hans-Peter Mock, Chiara Tonelli, Katia Petroni

**Affiliations:** 1 Dipartimento di Bioscienze, Università degli Studi di Milano, Milan, Italy; 2 Laboratory of Clinical Chemistry, Hospital of Varese, ASST-Settelaghi, Varese, Italy; 3 Laboratory of Clinical Pathology and Human Genetics, Foundation IRCCS Carlo Besta, Milan, Italy; 4 Leibniz Institute of Plant Genetics and Crop Plant Research (IPK), Gatersleben, Germany; 5 Present Address: School of Agriculture, Food and Wine, University of Adelaide, Adelaide, Australia

**Keywords:** Adipose tissue, Obesity, Inflammation, Anthocyanins

## Abstract

**Background:**

Obesity is a chronic and systemic inflammatory disorder and an important risk factor for the onset of several chronic syndromes. Adipose tissue (AT) plays a crucial role in the development of obesity, promoting the infiltration and accumulation of leukocytes in the tissue and sustaining adipocyte expansion. Anthocyanins exert a broad range of health benefits, but their effect in improving obesity-related inflammation in vivo has been poorly characterized. We examined the effects of a purple corn cob extract in the context of AT inflammation in a murine diet-induced obesity (DIO) model.

**Methods:**

Male C57BL/6J mice were subjected to control diet (CTR + H_2_O), high fat diet (HF + H_2_O) or high fat diet plus purple corn extract (HF + RED) for 12 weeks. Blood glucose, AT, and liver gene expression, metabolism, biochemistry, and histology were analysed and flow cytometry was performed on AT leukocytes and Kupffer cells.

**Results:**

RED extract intake resulted in lower MCP-1 mediated recruitment and proliferation of macrophages into crown-like structures in the AT. AT macrophages (ATM) of HF + RED group upregulated M2 markers (*ArgI*, *Fizz1*, *TGFβ*), downregulating inflammatory mediators (*TNF*-*α*, *IL*-*6*, *IL*-*1β*, *COX*-*2*) thanks to the suppression of NF-kB signalling. ATM also increased the expression of iron metabolism-related genes (*FABP4*, *Hmox1*, *Ferroportin*, *CD163*, *TfR1*, *Ceruloplasmin*, *FtL1*, *FtH1*) associated with a reduction in iron storage and increased turnover. ATM from HF + RED mice did not respond to LPS treatment ex vivo, confirming the long-lasting effects of the treatment on M2 polarization. Adipocytes of HF + RED group improved lipid metabolism and displayed a lower inflammation grade. Liver histology revealed a remarkable reduction of steatosis in the HF + RED group, and Kupffer cell profiling displayed a marked switch towards the M2 phenotype.

**Conclusions:**

RED extract attenuated AT inflammation in vivo, with a long-lasting reprogramming of ATM and adipocyte profiles towards the anti-inflammatory phenotype, therefore representing a valuable supplement in the context of obesity-associated disorders.

**Electronic supplementary material:**

The online version of this article (10.1186/s12967-019-1972-6) contains supplementary material, which is available to authorized users.

## Background

Obesity is a major metabolic disorder characterized by excess adipose tissue (AT), which represents a crucial risk factor for type 2 diabetes, cardiovascular diseases, cancer, and is associated with increased mortality [1, 2]. AT represents the largest endocrine organ in the body, which releases cytokines and adipokines into the circulation [3, 4], altering many physiological processes including triglycerides storage, thermogenesis, and insulin sensitivity [5]. Adipocytes are the main cellular components of AT [6], and in the obese phenotype they are usually characterized by hyperplasia and hypertrophy which impairs tissue functionality [7], developing apoptosis and becoming irresponsive to insulin. The chronic low-grade inflammation found in obesity is mostly characterised by higher levels of circulating pro-inflammatory cytokines and fatty acids (FA) which impact on insulin sensitivity as well as on the AT leukocyte ratio and phenotype, thus exacerbating the inflammatory milieu within the AT.

Macrophages are the main leukocyte population within AT and the major trigger of obesity-related inflammation [8]. In obese individuals, adipose tissue macrophages (ATM) are typically classically activated M1 cells found in aggregates characterized by a pro-inflammatory signature (*TNF*-*α*, *IL*-*6*, *IL*-*12*, *NO*, *iNOS*) promoting insulin resistance, while in lean subjects they are dispersed in the tissue, exhibit an M2 anti-inflammatory phenotype (*ArgI*, *Fizz1*, *Ym1*, *Mrc1*, *TGF*-*β* and *IL*-*10* expression), and support insulin sensitivity [9].

Anthocyanins (ACNs) are polyphenols found in pigmented plants, widely acknowledged for their anti-inflammatory, antioxidant, and anti-carcinogenic properties. Positive effects of anthocyanins have been described in epidemiological, clinical, and pre-clinical studies, and include reduction of LDL cholesterol, myocardial infarction, and cardiovascular diseases [10–12]. A number of studies showed that ACN-enriched diets can prevent obesity and insulin resistance [13, 14].

Using combinations of the *MYB* and *bHLH* anthocyanin regulatory genes, we previously generated purple corn with high ACN content in seeds and cobs similar to Andean purple corn, but adapted to the European climate and suitable for nutrigenomic studies [11, 15]. Purple corn mainly contains cyanidin 3-glucoside and, to a small extent, pelargonidin 3-glucoside and peonidin 3-glucoside [11, 16].

Purple corn cobs are normally wasted, despite their high ACN content, which could make them a cost-effective alternative source of anthocyanins for food supplements.

In this study, we explored the possibility of employing a purple corn cob extract as a natural and convenient anti-obesity and anti-inflammatory functional food supplement by assessing its in vivo effect in the context of AT inflammation in a murine diet-induced obesity (DIO) model.

## Methods

### Animals and diets

Eight-week old male C57BL/6J mice (n = 64 Charles River, Calco, Italy), were used and maintained at 23 ± 3 °C, 12 h light cycle (08.00–20.00). The sample size was determined a priori using a power analysis in order to reach 80% power at an alpha-level of 0.10 to detect a strong effect size considering effects reported in previous research [10, 11, 14]. Mice were randomly divided into 3 groups and assigned to either control diet (20% protein, 70% carbohydrate, and 10% fat; 3.85 kcal/g; #D12450B, Research Diets; New Brunswick, NJ, USA) or HF diet (20% protein, 35% carbohydrate and 45% fat; 4.73 kcal/g; #D12451, Research Diets) for 12 weeks along with water or RED extract supplied in drinking water at an ACN concentration of 290 mg/Kg body weight/day. Diets and drinks were replaced weekly and daily respectively, to prevent oxidization. Mice were weighed weekly and daily, food and water/RED extract intakes were recorded throughout the study. The RED extract was produced by SVEBA Srl (Appiano Gentile, Italy) to a final concentration of 40 mg/g of ACNs, as previously described [11]. After 12 weeks, liver, spleen, and epididymal adipose tissue were carefully removed and weighed. All procedures involving animals and their care conformed to institutional guidelines in compliance with national (D.L. N.116, G.U., suppl. 40, 18-2-1992; D.L. N. 26, G.U. 4-3-2014) and international law and policies (EEC Council Directive 2010/63/EU, OJ L 276/33, 22-09-2010; NIH Guide for the Care and Use of Laboratory Animals, US National Research Council-2011) and approved by the University of Milan Animal Welfare Body and by the Italian Minister of Health (Project number 7/2013). All efforts were made to minimize animal suffering and to reduce the number of animals used.

### HPLC and MS analysis of corn cob powder

Powdered plant material (100 mg of corn cob) was extracted twice with 400 µl of 80% MeOH with 2% formic acid. Prior to the injection 100 µl of extract were mixed with 25 µl of solvent A (water with 0.5% formic acid). After centrifugation 5 µl of mixture were injected per sample. Phenylpropanoids were separated by reverse-phase HPLC (Acquity UPLC™ with a CSH Phenyl-Hexyl, 1.7 µm, 2.1 × 100 mm column; Waters, Eschborn, Germany) at 25 °C. At a flow rate of 0.5 ml/min, the following gradient was applied: initial 2% solvent B (acetonitrile with 0.5% formic acid); 0.6 min, 6% B; 8 min, 20% B; 10 min, 40% B; 11 min, 95% B; 13 min, 95% B; 13.5 min, 2% B. Eluted substances were detected with a photodiode array detector (PDA 2996, Waters). Absorbance spectra were recorded at a frequency of 10 s^−1^ between 210 and 600 nm, with a bandwidth of 1.2 nm, and chromatograms were extracted from the PDA data at 535 nm. The outlet of the PDA detector was coupled online with an LCT Premier Time-of-Flight (TOF) mass spectrometer (Waters) equipped with an electrospray ionization (ESI) and modular LockSpray interface. The capillary voltage was 2.2 kV and the source temperature was 100 °C. Spectra were recorded in positive-ion W-mode between mass and charge ratios of 100 and 1000. Mass calibration was performed in the range of 100–1000 *m/z* with phosphoric acid. Leucine enkephalin (2 ng/μl in 50% (v/v) aqueous acetonitrile) was employed during lockspray operation as internal mass reference for automated accurate mass measurement. Data were analyzed using Waters Mass Lynx software. Tentative annotations in the analytical chromatographic runs are based on detected protonated molecular mass, in source fragment masses relating to the anthocyanidin aglycones and the retention time order (Additional file 1: Figure S1, Additional file 2: Table S1). Isolation and mass spectrometric identification of the major anthocyanins from purple cob powder was realized as previously described [11].

### Glucose tolerance tests, serum cholesterol and alanine aminotransferase quantification

For the glucose tolerance test (GTT), mice were fasted for 16 h, followed by an intraperitoneal glucose injection (2 g/kg body weight). Blood glucose was measured by tail bleeding using the One-Touch AccuChek Glucometer (Roche, Monza, Italy) at indicated times (0, 30, 60, 120 min).

Blood was collected in tubes containing 0.5 M EDTA, centrifuged at 1000×*g* for 15 min at 4 °C, and the plasma collected and stored at − 80 °C until further analysis. HDL and LDL/VLDL cholesterol and alanine aminotransferase (ALT) activity were determined in serum by colorimetric assay (Abcam Cambridge, UK), according to the manufacturer’s instructions. For total cholesterol levels, plasma aliquots (100 μl) were added to a screw-capped vial sealed with a Teflon septum together with epicoprostanol 250 μg as internal standard, butylated hydroxytoluene (BHT) and EDTA. Alkaline hydrolysis was allowed to proceed at room temperature (22 °C) with magnetic stirring for 30 min in the presence of ethanolic 0.5 M potassium hydroxide solution. Cholesterol was collected by liquid to liquid extraction with 5 ml of hexane. The organic solvents were evaporated under a gentle stream of argon and converted into trimethylsilyl ethers with BSTFA with TCS 1% (Pierce). Gas chromatography mass spectrometry (GC–MS) analysis was performed on an Elite column (30 m × 0.32 mm id × 0.25 mm film; Perkin Elmer, USA) and injection was performed in splitless mode and using helium (1 ml/min) as a carrier gas. The temperature program was as follows: initial temperature of 2000 °C was held for 1 min, followed by a linear ramp of 20 °C/min to 270 °C, 5 °C/min to 290 °C thus held for 8 min. The mass spectrometer operates in selected ion-monitoring mode. Cholesterol and epicoprostanol were monitored m/z 369 and m/z 371.

For determination of non-esterified fatty acids (NEFA), plasma aliquots (50 μl) were added to a screw-capped vial sealed with a Teflon septum together with 12.5 µg of pentadecanoic and 12.5 µg of hexadecanoic acid as internal standards, butylated hydroxytoluene (BHT), K3-EDTA and 600 µl of methanol. After vigorous vortexing and centrifugation, the methanol phase was collected and evaporated under a gentle stream of nitrogen and converted into trimethylsilyl ethers with BSTFA with TCS 1% (Pierce).

Gas chromatography mass spectrometry (GC–MS) analysis was performed on an Elite column (30 m × 0.32 mm id × 0.25 mm film; Perkin Elmer, USA) and injection was performed in splitless mode and using helium (1 ml/min) as a carrier gas. The temperature program was as follows: initial temperature of 140 °C was held for 1 min, followed by a linear ramp of 10 °C/min to 290 °C, and thus held for 3 min. The mass spectrometer operates in full scan (from m/z 50 up to m/z 550) mode [17].

### Isolation of peritoneal macrophages

Peritoneal elicited macrophages (PEC) were derived from mice intraperitoneally (i.p.) injected with 1 ml thioglycollate (3% thioglycollate medium w/o dextrose; Becton–Dickinson, Franklin Lakes, NJ, USA). After 4 days, cells were collected by washing the peritoneal cavity with 5 ml of ice-cold sterile PBS, incubated in erythrocyte lysis buffer for 5 min, washed and cultured in a humidified incubator containing 5% CO_2_ at 37 °C in 10% fetal calf serum (FCS)-RPMI 1640 medium for 24 h before treatment with extracts.

### Murine tissues collection and processing

Epididymal white adipose tissue (WAT) and livers were minced into fine (< 10 mg) pieces, placed in HEPES buffered DMEM (Thermo Fisher, Walthan, MA, USA) supplemented with 10 mg/ml BSA (Sigma-Aldrich, Milano, Italy), and centrifuged at 1000×*g* for 10 min at room temperature (RT). An LPS-depleted collagenase Type II cocktail (Sigma-Aldrich) at a concentration of 0.03 mg/ml and 50 U/ml DNase I (Sigma-Aldrich) was added to the tissue suspension, and the samples were incubated at 37 °C on an orbital shaker (215 Hz) for 45 min. Samples were then passed through a sterile 100 µm nylon mesh (Corning, NY, USA) and centrifuged at 1000×*g* for 10 min. The pelleted cells from the WAT were collected as the SVF and the floating cells as the adipocyte-enriched fraction. Cells were resuspended in erythrocyte lysis buffer for 5 min and centrifuged at 500×*g* for 5 min before proceeding with the analysis.

### Flow cytometry

Flow cytometry was performed using a BD FACSCantoII™ flow cytometer and analysis run with FACSDiva 6.1.1 software (Becton–Dickinson). Cells were stained with anti-mouse CD45-PB (#103125), CD11b-PE/Cy5 (#101209), F4/80-APC (#123115), F4/80-FITC (#123107), CD206-FITC (#141703), CD80-PE (#104707), SiglecF1-PE (#142403), Gr1-APC/Cy7 (#108423), CD3-APC (#100235), CD4-PE (#100407), CD8-PE/Cy5 (#100709) (BioLegend, San Diego, CA, USA) for 20 min at 4 °C, washed and resuspended in FACS Buffer (PBS, 5 mM EDTA and 0.2% BSA).

Intracellular staining for Ki67-AF488 (#561165, BD Biosciences) was performed by adding 3 ml cold 70% ethanol to the cell pellet and incubating at − 20 °C for 1 h, followed by staining with the antibody at RT for 30 min.

### Ex vivo cell treatment

Once isolated, PEC or SVF from C57BL/6J were pre-treated with the extracts at a concentration of 125 μM of RED extract or cyanidin 3-glucoside (C3G) (Extrasynthese, Genay, France) for 24 h. The cells where then challenged with 100 ng/ml LPS for an additional 4 h for mRNA collection or 24 h for supernatant collection.

### Analysis of gene expression

Total RNA was isolated from tissues and cells with Direct-Zol™ RNA MiniPrep kit (Zymo Research, Irvine, CA, USA). 1 μg RNA was reverse-transcribed with miScript II RT Kit (Qiagen, Hilden, Germany) and qRT-PCR performed with Fast Evagreen qPCR Master Mix (Bio-Rad, Segrate, Italy) in a CFX96 real-time PCR detection system (Bio-Rad). The expression of each specific mRNA was normalised against *GAPDH* and values were expressed as Fold Change on CTR + H_2_O sample. Values represent mean ± SEM obtained from duplicate measurements performed on 4–6 biological replicates, as indicated in Figure legends (n = 4–6 mice/group). Primer sequences are summarized in Additional file 2: Table S2.

### Enzyme-linked immunosorbent assay (ELISA)

Samples were analysed for mouse IL-6, mouse IL-1β, and mouse TNF-α using Ready SET-Go! ELISA kit (Affymetrix, Santa Clara, CA, USA). The optical density (OD) of each well was analysed at 450 nm by a Microplate Reader (Tecan Infinite F200PRO).

### Western blot analysis

Adipose tissues were lysed in a buffer containing 50 mM Tris–HCl, pH 7.2, 0.1% sodium deoxycholate, 1% Triton X-100, 5 mM EDTA, 5 mM EGTA, 150 mM NaCl, 40 mM NaF, 2.175 mM NaVO_4_, 0.1% SDS, 0.1% aprotinin, and 1 mM PMSF. 30 μg of protein underwent SDS–PAGE following transfer on nitrocellulose membranes. Bands were detected using Pierce™ ECL Western Blotting Substrate (ThermoFisher Scientific). Antibodies against Anti-NF-kB p65 (Abcam, ab86299) and α-Tubulin (Sigma, T9026) were used. Densitometry quantification was performed with ImageJ Software (NIH) and expressed as ratio of specific protein to α-tubulin.

### Histochemistry and prussian blue-DAB iron staining

After fixing in 4% paraformaldehyde overnight, the fat and livers were dehydrated and embedded in paraffin. 5 μm sections were deparaffinised and incubated for 40 min in 2% HCl containing 10% potassium ferrocyanide, washed in 0.1 M phosphate buffer, followed by 0.025% 3,3′-DAB-4HCl (DAB; Sigma-Aldrich) and 0.005% H_2_O_2_ in a 0.1 M phosphate buffer for 40 min and/or counterstained with haematoxylin and eosin (H&E; Carl Roth, Karslruhe, Germany) and observed under a light microscope (Leica DM6000B, Wetzlar, Germany). For quantitative analysis of adipocyte area, five images per section were randomly acquired from each sample, and the cross-sectional area of each adipocyte was measured using ImageJ software (NIH).

### Statistical analysis

All statistical analyses were performed using GraphPad Prism 6.0 software. Data are presented as mean ± SEM, and the numbers of independent experiments are indicated for each data set. For statistical analysis, basal versus LPS-treated samples were compared using unpaired two-tailed student’s *t*-tests, whereas the three dietary groups were compared by one-way ANOVA followed by Bonferroni post hoc tests for multiple comparisons using GraphPad Prism 6.0 software. p < 0.05 was considered statistically significant. The level of significance is indicated by asterisks (****p < 0.0001; ***p < 0.001; **p < 0.01; and *p < 0.05). Experiments were performed in triplicate.

## Results

### Body weight, adipose tissue and liver weight and serum cholesterol

**Fig. 1:**
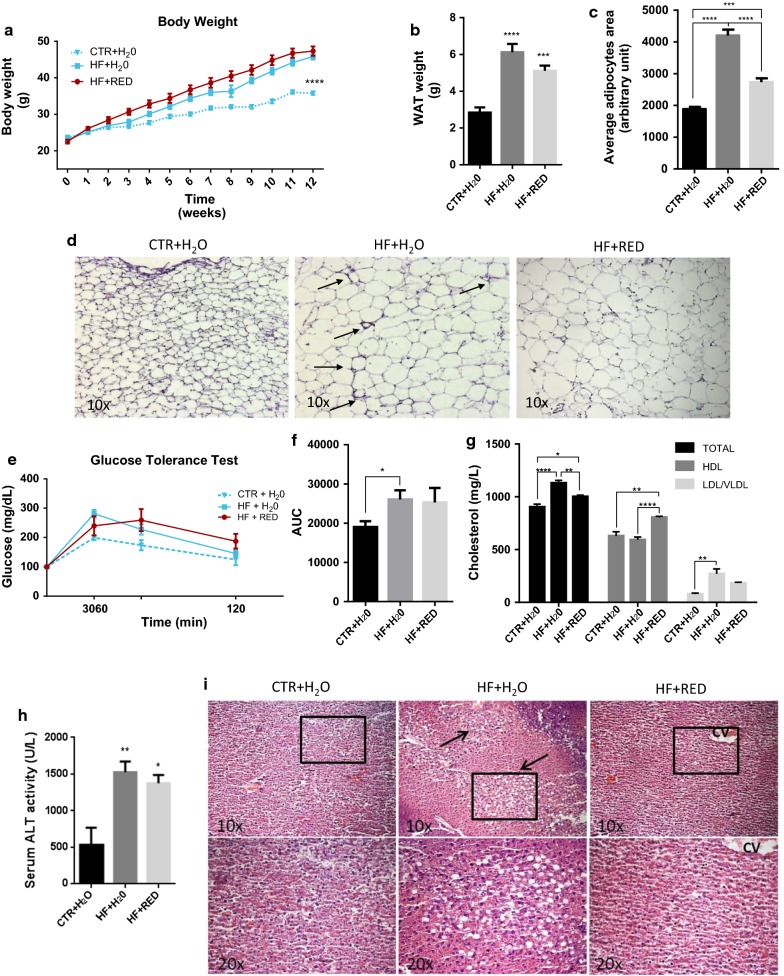
Fat weight, adipocyte area, serum cholesterol and hepatosteatosis in DIO mice. **a** Body weight gain over time; **b** epididymal WAT weight at week 12; **c** mean adipocyte area. **d** Representative images of H&E-stained sections of epididymal WAT at ×10; arrows indicate CLS. Each image is representative of 6 mice. **e** Glucose tolerance test (GTT) and **f** AUC after 16 h fasting (n = 10). **g** Total, HDL and LDL/VLDL cholesterol and **h** ALT activity. Mean ± SEM, n = 10. **i** H&E staining of liver sections from representative mice from each group with ×10 magnification (upper panel) and with ×20 magnification (lower panel); CV: lobular central vein. Each image is representative of 6 mice. Arrows indicate areas with lipid droplets deposition. AUC, area under the curve. ALT, alanine aminotransferase

C57BL/6J male mice were maintained on CTR + H_2_O, HF + H_2_O or HF + RED for 12 weeks. At the time of harvest, the weight of both HF groups was significantly greater than that of the CTR + H_2_O (Fig. 1a). No differences were noticed between the two HF groups. Liquid intake was comparable among the three groups throughout the entire experiment (Additional file 1: Figure S2A). Food intake was slightly lower in the two HF groups compared with the control group (Additional file 1: Figure S2B), but it did not differ between the HF + H_2_O and HF + RED groups. Energy intake was constant throughout the experiment and feed efficiency was greater in the two HF groups compared to CTR + H_2_O (Additional file 2: Table S3). WAT weight was significantly greater in the two HF groups than in the control (2.8 ± 0.28 g, 7.5% of total body mass) and, although not statistically significant, greater in the HF + H_2_O (6.117 ± 0.45 g, 13.2% of total body mass) compared to HF + RED (5.104 ± 0.28 g, 10.6% total body mass) (Fig. 1b). Histological examination of the WAT revealed a marked reduction in adipocyte cell size area in the HF + RED group (2735 ± 119 μm) compared to HF + H_2_0 (4203 ± 183 μm) (Fig. 1c, d), suggesting adipocyte hypertrophy likely accounts for the increase in total adiposity. Blood glucose levels were recorded at time 0, 30, 60, and 120 min after injection of glucose to evaluate changes in glucose tolerance (Fig. 1e). In the HF + H_2_O group the relative area under the curve (AUC) was increased by 36.8% (p = 0.0048), while the HF + RED group showed an increase of the 32.8% (p = 0.1981; Fig. 1f) relatively to the control group. Total and LDL/VLDL cholesterol levels were increased in both HF groups compared to CTR + H_2_O and significantly reduced in HF + RED compared to HF + H_2_O, whereas HDL cholesterol was significantly increased in HF + RED compared to both CTR + H_2_O and HF + H_2_O (Fig. 1g). No difference in total NEFA was observed in the three groups, despite a modest reduction in miristic, palmitic, linoleic, oleic and stearic acids in HF + RED compared to HF + H_2_O (Additional file 2: Table S4). Serum ALT activity was significantly greater in the two HF groups than in CTR + H_2_O, with a modest, but still not significant, reduction in HF + RED compared to HF + H_2_O (Fig. 1h). Liver weight did not change among the three groups (data not shown), however HF + H_2_O mice liver sections stained with H&E displayed abundant steatosis compared to the CTR + H_2_O as well as HF + RED, indicating that the purple corn extract might inhibit fat accumulation in hepatocytes (Fig. 1i).

### RED extract ameliorates obesity-associated immunological profile of WAT

**Fig. 2:**
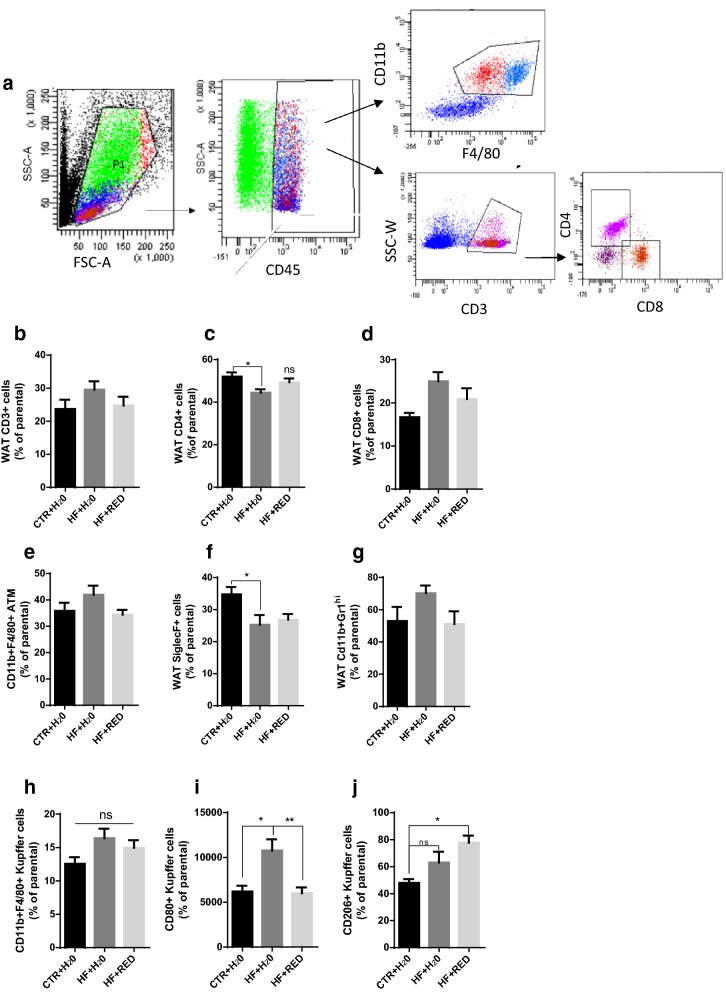
Flow cytometric analysis of epididymal WAT leukocytes and Kupffer cells. **a** Gating strategy. **b**–**j** Immunostaining for markers of T cells (CD3, CD4, and CD8, **b**–**d**), ATM and Kupffer cells (CD11b and F4/80, **e**, **h**), eosinophils (Siglec-F, **f**), and neutrophils (Gr1, **g**). **i** M1 Kupffer cells are identified by CD80 expression. **j** M2 Kupffer cells are identified by CD206 expression. Mean ± SEM (n = 10–12 mice/group)

The SVF from epididymal WAT was stained with antibodies to assess leukocyte populations by flow cytometry. Macrophages were defined as F4/80^+^/CD11b^+^/Siglec-F^−^/Gr-1^−^ cells (Fig. 2a). Notably, CD3^+^ T cells accounted for approximately 24% of the CD45^+^ cells in both control and HF + RED groups, while set around 30% in the HF + H_2_O group (Fig. 2b). Moreover, despite the overall prevalence of CD4^+^ T cells in all three groups (Fig. 2c), the CD4^+^ T cell fraction was larger in CTR + H_2_O (52%) and HF + RED (50%) groups than in the HF + H_2_O group (44%), whereas 25% of the CD3^+^ T cells in the HF + H_2_O group were CD8^+^ , against the 20% in HF + RED and 16% in CTR + H_2_O (Fig. 2d) groups, resulting in a slightly improved CD8/CD4 cell ratio of the HF + RED group compared to HF + H_2_O group (Additional file 1: Figure S3).

Macrophage percentage was slightly higher in the HF + H_2_O group (42%) compared to CTR + H_2_O (35%) and HF + RED (34.4%) (Fig. 2e). Eosinophil content in the AT diminishes in obesity, as confirmed in both HF + H_2_O (25.2%), and HF + RED (26%) groups, compared to CTR + H_2_O (35%) (Fig. 2f). Neutrophil content in AT was higher in the HF + H_2_O group compared to CTR + H_2_O and HF + RED groups (Fig. 2g), although the difference was not significant.

F4/80 and CD11b were used to identify resident hepatic Kupffer cells (Fig. 2h–j). As shown in Fig. 2h, the Kupffer cell populations in the liver were not significantly different among the three groups, in agreement with Clementi et al. [18]. However, analysis revealed a reduction in CD80^+^ pro-inflammatory macrophages in the HF + RED group (Fig. 2i) compared to the HF + H_2_O group, along with an increase in the number of CD206^+^ anti-inflammatory macrophages (Fig. 2j), suggesting that the RED extract might have the potential to modulate the liver’s contribution to the inflammatory state of obesity.

### RED extract reduces the recruitment and proliferation of CLS macrophages and promotes alternative phenotype switch

**Fig. 3:**
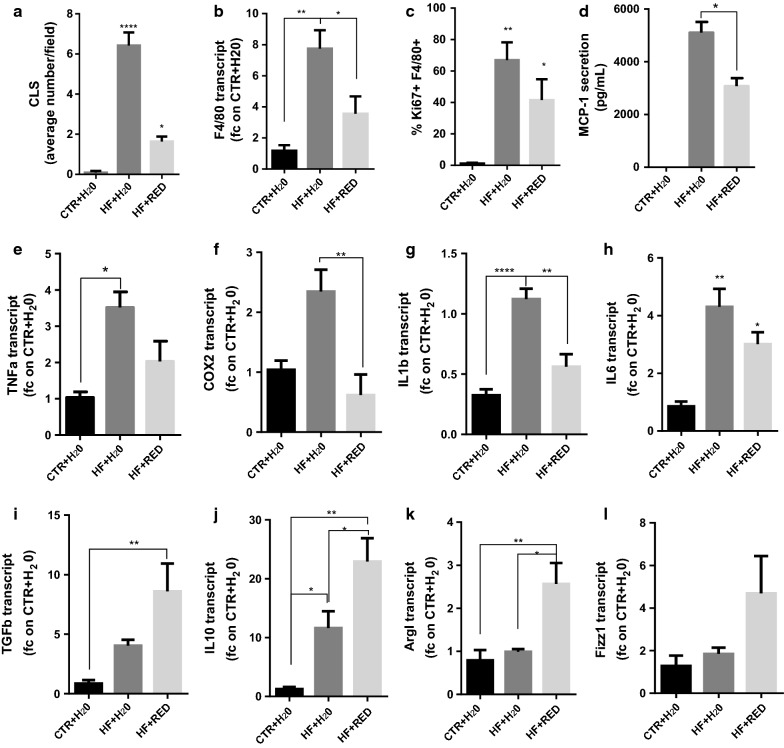
Macrophage phenotype, recruitment and proliferation and MCP-1 secretion in epididymal WAT. **a** Crown like structures (CLS) density in H&E sections. **b** qRT-PCR analysis of macrophage marker F4/80 expression in the WAT. **c** Intracellular staining for Ki67. **d** MCP-1 ELISA in WAT homogenate. **e**–**l** qRT-PCR analysis for gene expression of M1 (**e**–**h**) and M2 macrophage markers (**i**–**l**) in ATM. Each transcript was normalized to that in CTR + H_2_O (n = 6 mice/group). Mean ± SEM. n = 5–6 mice/group

Obesity triggers the formation of crown-like structures (CLS) around individual adipocytes, typical of localized chronic inflammation [19]. We characterized the epididymal WAT histology in H&E sections. We observed a predominance of macrophages aggregated into ring patterned CLS in the HF + H_2_O (6.417 ± 0.6566) group compared to the HF + RED (1.636 ± 0.2439) (Fig. 3a). No CLS were observed in the control group, where ATM were rather dispersed throughout the tissue. The decrease in HF + RED ATM was also confirmed by the reduced expression of the macrophage marker F4/80 in the epididymal WAT (Fig. 3b).

We therefore tested by flow cytometry whether ATM proliferation was reduced upon RED extract administration by staining SVF cells with the proliferation marker Ki67 and F4/80. Ki67 signal was detected in 66.94 ± 11.31% of ATM from the HF + H_2_O group, against 41.50 ± 13.27% of HF + RED and only 1.240 ± 0.49% of CTR + H_2_O (Fig. 3c), confirming a discrete reduction of the proliferation upon RED extract administration.

In order to characterize whether the decrease in ATM was due to a reduction in the monocyte chemoattractant protein-1 MCP-1 secretion by the tissue, we performed ELISA on epididymal WAT. As shown in Fig. 3d, the HF + RED group expresses significantly less MCP-1 (3076 ± 305.6 pg/ml) compared to the HF + H_2_O group (5105 ± 404.2 pg/ml), which is consistent with a less inflammatory milieu.

We next assessed the expression of a panel of pro- and anti-inflammatory genes that represent markers of M1 and M2 macrophages respectively, to test the potential of the RED extract in promoting an ATM phenotypic switch in vivo. qRT-PCR analysis from ATM revealed that expression of all M1 inflammatory genes (*TNF*-*α*, *COX*-*2*, *IL*-*1β*, and *IL*-*6*) was dramatically downregulated in the ATM of the HF + RED group at levels comparable to those of the CTR + H_2_O group (Fig. 3e–h). In addition, RED extract consumption was associated with an upregulation in the expression of all the alternative polarization markers (*TGF*-*β*, *IL*-*10*, *ArgI*, and *FizzI*) (Fig. 3i–l), confirming the results obtained in vitro of an anti-inflammatory effect of the RED extract on ATM due to its ability to induce an M2 phenotype switch.

### ATM from RED extract diet display an iron recycling profile

Macrophages play a pivotal role in systemic iron recycling [20], contributing to adipogenesis and AT homeostasis. AT iron distribution was assessed by Perls’ Prussian blue method. Most of the iron depots within the AT were found coincidentally with CLS surrounding adipocytes in the HF + H_2_O group, whereas lower iron staining was detected in HF + RED sections (Additional file 1: Figure S4A). This evidence was further confirmed by qRT-PCR analysis of the genes involved in iron metabolism. The HF + RED group displayed an increase in all iron metabolism genes (Additional file 1: Figure S4B–G), and a downregulation of the iron-storage gene *FtL1* (Additional file 1: Figure S4H), consistent with an iron recycling phenotype.

### RED extract improves adipocyte metabolism through the modulation of NF-kB pathway

We investigated whether our previous histological and molecular findings of the anti-inflammatory effect of RED extract on ATM also translates to a better adipocyte metabolic profile.

**Fig. 4:**
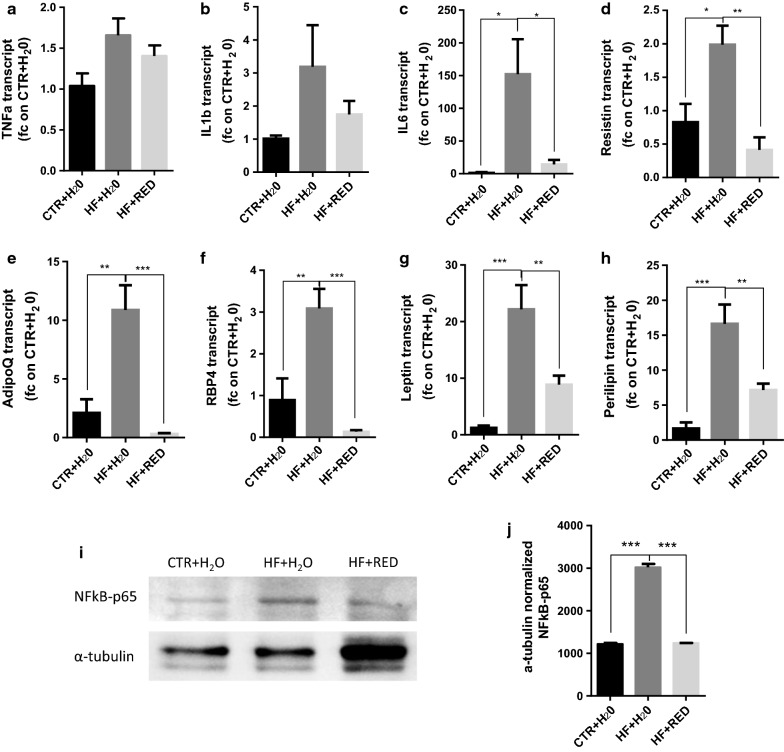
Adipokines and metabolic genes expression in adipocytes. **a**–**h** qRT-PCR for the expression of prototypical adipokines in adipocytes. Each transcript was normalized to that in CTR + H_2_O. **i** Western blot panel and **j** relative quantification of NF-kB-p65 levels. Data have been analysed with ImageJ and normalized to α-tubulin. Mean ± SEM. n = 6 mice/group

In adipocytes from epididymal AT, the most common adipokines (*TNF*-*α*, *IL*-*6*, *IL*-*1β, resistin*, *RBP4*, *leptin*, *adiponectin*, and *perilipin*), were all downregulated in the HF + RED condition compared to the HF + H_2_O one (Fig. 4a–h). To investigate whether the inhibitory effect of the RED extract on pro-inflammatory genes and adipokine expression relates to transcription factor NF-κB, the nuclear levels of NF-κB p65 were determined in epididymal AT lysates by Western blot analysis. Despite the increased expression of α-tubulin observed in HF + RED condition, when normalized to α-tubulin, NF-κB p65 expression was significantly inhibited in the HF + RED sample compared to the HF + H_2_O group after only 12 weeks of extract consumption (Fig. 4i, j), and resembled that of the untreated control group.

### RED extract protects obese mice from inflammatory response to LPS

**Fig. 5:**
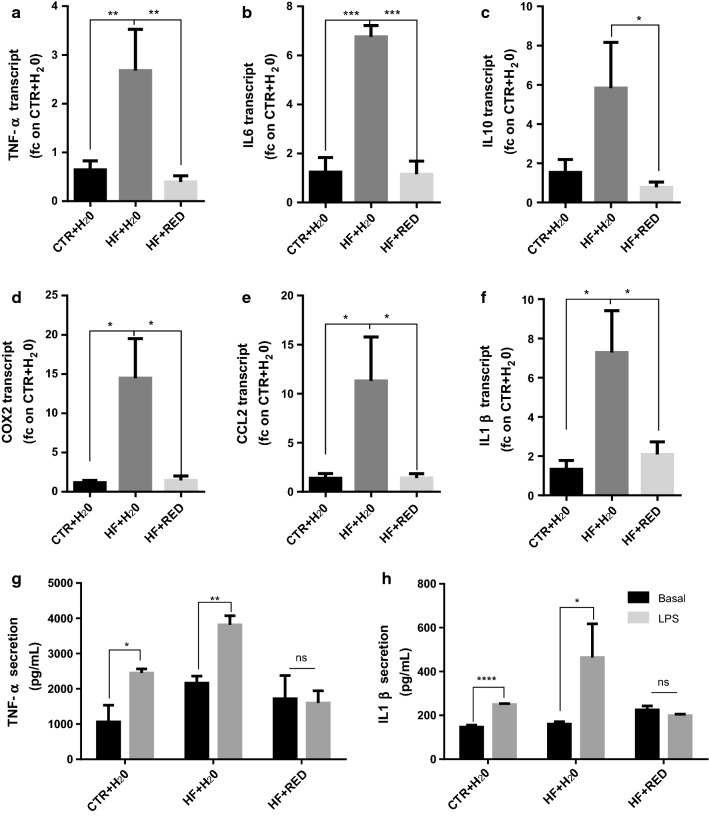
LPS-induced inflammatory response in vivo. **a**–**f** qRT-PCR for the expression of genes involved in LPS response. SFV was isolated from AT and plated in 12-well plates following exposure to 100 ng/mL LPS for 4 h. Each transcript was normalized to that in CTR + H_2_O group (n = 6 mice/group). **g**, **h** ELISA for TNF-α and IL-1β of the supernatant of SFV from the three groups upon ex vivo exposure to LPS for 24 h. Mean ± SEM. n = 4 mice/group

Given obesity is characterized by a low-grade inflammation, we investigated whether the RED extract consumption was able to exert a protective effect in vivo similar to the one observed in vitro (Additional file 1: Figure S5). SVF from the epididymal AT was exposed to 100 ng/ml LPS for 4 h. LPS-induced inflammatory cytokine expression was significantly suppressed by RED extract consumption (Fig. 5a–f). Next, we used ELISA assay to determine the production of TNF-α and IL-1β upon 24 h LPS. The data showed that cytokine production was significantly suppressed in the RED extract samples compared to both HF + H_2_O and CTR + H_2_O upon LPS treatment (Fig. 5g, h), confirming that the RED extract consumption indeed retains its protective effects also in vivo.

## Discussion

Recently, great attention has focused on the anti-obesity properties of bioactive food components, particularly anthocyanins and polyphenols [13]. Purple corn cob contains a very high concentration of anthocyanins [11] compared to other anthocyanin-rich sources [21]. Many health benefits have been associated with purple corn, including reduction of drug-induced cardiotoxicity and trigeminal inflammation [11, 22], prevention of obesity and diabetes [14] and reduction of TNF-α-induced inflammation in adipocytes in vitro [23].

Our results demonstrated that in PEC the RED extract remarkably decreased the production of all the main pro-inflammatory cytokines analysed, at the same, or even higher, extent than the pure C3G (Additional file 1: Figure S5).

Since it was particularly effective in dampening inflammation in vitro, we speculated that the RED extract could ameliorate HF diet-induced chronic low-grade AT inflammation also in vivo.

Although our 12-weeks treatment failed to reduce the animals’ total body mass, epididymal WAT measurement and histological analysis of the AT revealed a modest reduction in epididymal fat weight, which only meets the generous standards of statistical significance (p = 0.06), along with a significant reduction in the average adipocyte area. The effects observed suggest an overall increase in lean mass, indicating that the RED extract might be effective against body weight gain long term.

Obesity-associated liver steatosis and plasma cholesterol level were reduced upon RED extract consumption, and the Kupffer cell profile shifted towards the M2 phenotype, suggesting an improvement also outside the AT [24]. Furthermore, our data indicate a trending reduction in most of the plasma NEFA analysed. High NEFA concentrations in the blood can indeed alter resolution of inflammation in obesity [25].

Macrophages are the largest and most representative immune population in the AT, accounting for the majority of the cytokine production [6, 9]. Several in vitro studies described how polyphenols have a direct impact on immune cell trafficking and infiltration [26, 27], phenotypic switch [28], and secretion of pro-inflammatory mediators [29], including the monocyte chemotactic protein-1 (MCP-1) [30]. This latter is at least partly responsible for the increased number of CLS and macrophage proliferative capacity in the AT observed in obese subjects. In our study, RED extract consumption feebly ameliorated the ratio of CD8/CD4 T cells (Additional file 1: Figure S3) normally higher in obesity [8, 9]. Most importantly, it significantly reduced the recruitment of macrophages into CLS within the AT. ATM in the HF + RED group appeared mostly isolated and characterized by a lower proliferation rate, probably as a consequence of the considerably lower production of MCP-1 in the AT.

The RED extract also normalized the ATM expression of the M1 pro-inflammatory genes (*TNF*-*α*, *COX*-*2*, *IL*-*1β*, and *IL*-*6*) while upregulating the expression of the M2 markers (*TGF*-*β*, *IL10*, *ArgI*, and *FizzI*), hence positively modifying the M1/M2 ratio in the AT.

Iron overload in AT is known to contribute to local and systemic insulin resistance [31], and obesity-related inflammation often leads to iron-deficient anaemia [32] due to the sequestration of iron within the tissue. Adipogenesis itself is associated with genes regulating iron metabolism [33]. Macrophages play the pivotal role in systemic iron recycling [20], contributing to AT homeostasis. In vitro studies on macrophage iron handling suggests that M2 polarization is associated with an iron-recycling phenotype characterized by elevated iron metabolism gene expression and an increased capacity for iron uptake and release, while M1 polarization elicits an iron sequestration phenotype [34]. Our findings of the increase of M1 cells with iron storage phenotype in the HF + H_2_O group are in line with a number of studies demonstrating how iron overload is associated with inflammatory disorders [35–37]. This condition was reverted by the administration of RED extract, which induced a shift towards M2/recycling phenotype, consistent with a change in ATM function within the tissue. Consistently with our results, an in vivo study from Orr et al. [38] confirmed that high fat diet (HFD) induces a shift in macrophages polarization toward an inflammatory phenotype and it impairs ATM and adipocytes iron handling, resulting in the decreased expression of genes involved in iron uptake, metabolism, storage and export [38]. In their setting, however, contrarily to our observations, M1 macrophages from HFD mice were characterized by a reduction in the iron content. The above discrepancy might derive from differences in experimental conditions, such as diet duration and composition, which might impact on the phenotype and function of the monocyte- macrophage population, characterized by huge heterogeneity.

Insulin sensitivity and glucose homeostasis of liver, skeletal muscle, pancreas, and central nervous system are all affected by the adipokines produced by adipocytes [39]. Hypertrophic adipocytes in obese AT upregulate expression of inflammatory genes and impair insulin responsiveness, leading to increased lipolysis and the toxic release of fatty acids. Based on the local crosstalk between ATM and adipocytes within the AT, we speculated that the change in ATM profile upon RED extract consumption may reflect also in an improvement in adipocyte metabolism. We demonstrated that the consumption of RED extract restored the adipocyte profile to that found in the control diet group, abolishing adiponectin and leptin resistance [40], and dampening the overexpression of all the genes associated with the development of insulin resistance [41, 42]. In humans, adiponectin levels are inversely, whereas adiponectin receptor levels are positively related to obesity, glucose, lipids and insulin resistance. However, in mice, decreased expression of adiponectin occurs only upon prolonged (18 weeks) chronic high fat feeding, following increase around week 10 [43]. The increased adiponectin levels we report could represent a compensatory mechanism by which mice attempt to prevent the onset of insulin resistance in early stages of high fat diet exposure.

NF-κB p65 is a major transcriptional activator for inflammatory genes, whose overexpression seems to be the source of the chronic inflammation found in obese AT, and can be downregulated in vitro by ACN supplementation [44]. Despite the increased α-tubulin expression observed in HF + RED dietary treatment, the relative quantification of NF-kB-p65 levels normalized to α-tubulin indicate a clear reduction in NF-kB expression upon RED consumption, suggesting that our treatment was able to counteract the overexpression of NF-kB induced by DIO, and revealing one possible mechanism behind the anti-inflammatory and anti-obesity effects exerted by the RED extract consumption on the ATM.

Our ex vivo studies proved that this effect was triggered mainly by ATM. Moreover, we established for the first time that the RED extract consumption does not reflect in a temporary ATM repolarization, but rather in a long-lasting cellular reprogramming affecting the inflammatory response, even when cells are isolated from their physiological microenvironment.

Of note, all these benefits associated with the RED extract consumption derive from a 3 months study, indicating that a long-term treatment might produce even more dramatic benefits.

Although further research is required, our results suggest that RED extract represents a promising adjuvant for the amelioration of obesity-related inflammatory disorders, accompanied by a cost-efficient production and no side effects. In fact, purple corn extract has proved its safety in a clinical trial on breast cancer patients undergoing radiotherapy [45].

## Conclusions

Here, we demonstrated that RED extract ameliorates AT inflammation in vivo, with a long-lasting reprogramming of ATM and adipocyte profiles towards the anti-inflammatory phenotype, therefore representing a valuable supplement against obesity-associated disorders.

## Additional files

**Additional file 1: Figure S1.** Characterization of the anthocyanin profile and content. Shown in red is a representative HPLC–UV chromatogram from purple corn cob powder at 535 nm detection wavelength. The black line indicates the respective chromatogram of a control sample. Peaks marked with numbers (1 to 7) represent the major anthocyanins whose spectral characteristics, molecular ions and fragments are listed in Additional file 2: Table S1. **Figure S2.** (A) Liquid intake and (B) food intake of mice fed control or HF diet with or without RED for 12 wk. Mean ± SEM, *n* = 10. **Figure S3.** Ratio of CD4^+^/CD8^+^ T cells as determined by immunostaining of WAT from mice fed CTR + H_2_O, HF + H_2_0 or HF + RED. **Figure S4.** (A) Perls’ Prussian blue staining with DAB intensification of epididymal WAT sections. (B-H) qRT-PCR for the expression of genes involved in iron metabolism. (*heme oxygenase*-*1*, *Hmox; ferroportin*-*1*, *Fpn1; transferrin receptor*-*1*, *TfR1; ferritin light*, *Ftl1 and heavy chains*, *Fth1*). Each transcript was normalized to that in CTR + H_2_O (n = 6 mice/group). Mean ± SEM. **Figure S5.** (A-F) qRT-PCR analysis of *TNF*-*α*, *IL*-*6*, *IL*-*10*, *COX2*, *CCL2*, *IL*-*1* in PEC cultured with anthocyanin (125 μM) for 24 h and LPS (100 ng/ml) for additional 4 h. (G-I) TNF-α, IL-6 and IL-1β secretion by macrophages primed or not with RED and C3G upon LPS stimulation for 24 h. Mean ± SEM (n = 4 mice/group).

**Additional file 2.** Additional tables.

## Data Availability

All data generated or analysed during this study are included in this published article and its additional files.

## Abbreviations

ACN: anthocyanin
ALT: alanine aminotransferase
Arg-1: arginase 1
AT: adipose tissue
ATM: AT macrophages
CLS: crown-like structures
COX-2: cyclooxygenase-2
DIO: diet-induced obesity
FA: fatty acids
FABP4: fatty acid binding protein 4
FtH1: ferritin heavy chain 1
FtL1: ferritin light polypeptide 1
GTT: glucose tolerance test
HF: high fat
Hmox1: heme oxygenase1
IL-1β: interleukin 1β
IL-6: interleukin 6
IL-10: interleukin 10
IL-12: interleukin 12
iNOS: inducible nitric oxide synthase
MCP-1: monocyte chemotactic protein-1
Mrc1: mannose receptor C-type 1
NEFA: non-esterified fatty acids
NF-kB: nuclear factor kappa-light-chain-enhancer of activated B cells
NO: nitric oxide
RBP4: Retinol Binding Protein 4
SVF: stromal vascular fraction
TfR1: transferrin receptor protein 1
TGF-β: transforming growth factor-β
TNF-α: tumor necrosis factor- α
WAT: white adipose tissue
Ym1: chitinase-like 3

## References

[1] Pi-Sunyer FX (1999). Comorbidities of overweight and obesity: current evidence and research issues. Med Sci Sports Exerc.

[2] Reilly JJ, Kelly J (2011). Long-term impact of overweight and obesity in childhood and adolescence on morbidity and premature mortality in adulthood: systematic review. Int J Obes.

[3] Trayhurn P, Wood IS (2004). Adipokines: inflammation and the pleiotropic role of white adipose tissue. Br J Nutr.

[4] Kern PA, Saghizadeh M, Ong JM, Bosch RJ, Deem R, Simsolo RB (1995). The expression of tumor necrosis factor in human adipose tissue. Regulation by obesity, weight loss, and relationship to lipoprotein lipase. J Clin Invest.

[5] Chatterjee K, Sen C (2014). Respiratory morbidity in obesity, beyond obstructive sleep apnea. Ann Thorac Med.

[6] Weisberg SP, McCann D, Desai M, Rosenbaum M, Leibel RL, Ferrante AW (2003). Obesity is associated with macrophage accumulation in adipose tissue. J Clin Invest..

[7] Jo J, Gavrilova O, Pack S, Jou W, Mullen S, Sumner AE (2009). Hypertrophy and/or hyperplasia: dynamics of adipose tissue growth. PLoS Comput Biol.

[8] Chawla A, Nguyen KD, Goh YP (2011). Macrophage-mediated inflammation in metabolic disease. Nat Rev Immunol.

[9] Lumeng CN, Bodzin JL, Saltiel AR (2007). Obesity induces a phenotypic switch in adipose tissue macrophage polarization. J Clin Invest..

[10] Toufektsian MC, de Lorgeril M, Nagy N, Salen P, Donati MB, Giordano L, Martin HP (2008). Chronic dietary intake of plant-derived anthocyanins protects the rat heart against ischemia-reperfusion injury. J Nutr.

[11] Petroni K, Trinei M, Fornari M, Calvenzani V, Marinelli A, Micheli LA (2017). Dietary cyanidin 3-glucoside from purple corn ameliorates doxorubicin-induced cardiotoxicity in mice. Nutr Metab Cardiovasc Dis..

[12] Wallace TC (2011). Anthocyanins in cardiovascular disease. Adv Nutr.

[13] Titta L, Trinei M, Stendardo M, Berniakovich I, Petroni K, Tonelli C (2010). Blood orange juice inhibits fat accumulation in mice. Int J Obes..

[14] Tsuda T, Horio F, Uchida K, Aoki H, Osawa T (2003). Dietary cyanidin 3-*O*-beta-d-glucoside-rich purple corn color prevents obesity and ameliorates hyperglycemia in mice. J Nutr.

[15] Petroni K, Pilu R, Tonelli C (2014). Anthocyanins in corn: a wealth of genes for human health. Planta.

[16] Pedreschi LC-Z (2007). Phenolic profiles of Andean purple corn (*Zea mays* L.). Food Chem.

[17] Leoni V, Caccia C (2013). 24S-hydroxycholesterol in plasma: a marker of cholesterol turnover in neurodegenerative diseases. Biochimie.

[18] Clementi AH, Gaudy AM, van Rooijen N, Pierce RH, Mooney RA (2009). Loss of Kupffer cells in diet-induced obesity is associated with increased hepatic steatosis, STAT3 signaling, and further decreases in insulin signaling. Biochim Biophys Acta.

[19] Cinti S, Mitchell G, Barbatelli G, Murano I, Ceresi E, Faloia E (2005). Adipocyte death defines macrophage localization and function in adipose tissue of obese mice and humans. J Lipid Res.

[20] Ganz T (2012). Macrophages and systemic iron homeostasis. J Innate Immun..

[21] Moyer RA, Hummer KE, Finn CE, Frei B, Wrolstad RE (2002). Anthocyanins, phenolics, and antioxidant capacity in diverse small fruits: vaccinium, rubus, and ribes. J Agric Food Chem.

[22] Magni G, Marinelli A, Riccio D, Lecca D, Tonelli C, Abbracchio MP, Petroni K (2018). Purple corn extract as anti-allodynic treatment for trigeminal pain: role of microglia. Front Cell Neurosci.

[23] Luna-Vital D, Weiss M, Gonzalez de Mejia E (2017). Anthocyanins from purple corn ameliorated tumor necrosis factor-α-induced inflammation and insulin resistance in 3T3-L1 adipocytes via activation of insulin signaling and enhanced GLUT4 translocation. Mol Nutr Food Res.

[24] Odegaard JI, Ricardo-Gonzalez RR, Red Eagle A, Vats D, Morel CR (2008). Alternative M2 activation of Kupffer cells by PPARdelta ameliorates obesity-induced insulin resistance. Cell Metab.

[25] Huang S, Rutkowsky JM, Snodgrass RG, Ono-Moore KD, Schneider DA, Newman JW (2012). Saturated fatty acids activate TLR-mediated proinflammatory signaling pathways. J Lipid Res.

[26] Kang MK, Li J, Kim JL, Gong JH, Kwak SN, Park JH (2012). Purple corn anthocyanins inhibit diabetes-associated glomerular monocyte activation and macrophage infiltration. Am J Physiol Renal Physiol.

[27] Peiffer DS, Wang LS, Zimmerman NP, Ransom BW, Carmella SG, Kuo CT (2016). Dietary consumption of black raspberries or their anthocyanin constituents alters innate immune cell trafficking in esophageal cancer. Cancer Immunol Res.

[28] Aharoni S, Lati Y, Aviram M, Fuhrman B (2015). Pomegranate juice polyphenols induce a phenotypic switch in macrophage polarization favoring a M2 anti-inflammatory state. BioFactors.

[29] Lee SG, Kim B, Yang Y, Pham TX, Park YK, Manatou J (2014). Berry anthocyanins suppress the expression and secretion of proinflammatory mediators in macrophages by inhibiting nuclear translocation of NF-kappaB independent of NRF2-mediated mechanism. J Nutr Biochem.

[30] Amano SU, Cohen JL, Vangala P, Tencerova M, Nicoloro SM, Yawe JC (2014). Local proliferation of macrophages contributes to obesity-associated adipose tissue inflammation. Cell Metab.

[31] Gabrielsen JS, Gao Y, Simcox JA, Huang J, Thorup D, Jones D (2012). Adipocyte iron regulates adiponectin and insulin sensitivity. J Clin Invest..

[32] Tussing-Humphreys LM, Nemeth E, Fantuzzi G, Freels S, Guzman G, Holterman AX, Braunschweig C (2010). Elevated systemic hepcidin and iron depletion in obese premenopausal females. Obesity.

[33] Festa M, Ricciardelli G, Mele G, Pietropaolo C, Ruffo A (2000). Overexpression of H ferritin and up-regulation of iron regulatory protein genes during differentiation of 3T3-L1 pre-adipocytes. J Biol Chem.

[34] Recalcati S, Locati M, Marini A, Santambrogio P, Zaninotto F, De Pizzol M (2010). Differential regulation of iron homeostasis during human macrophage polarized activation. Eur J Immunol.

[35] Yang F, Liu XB, Quinones M, Melby PC, Ghio A, Haile DJ (2002). Regulation of reticuloendothelial iron transporter MTP1 (Slc11a3) by inflammation. J Biol Chem.

[36] Schaer CA, Vallelian F, Imhof A, Schoedon G, Schaer DJ (2008). Heme carrier protein (HCP-1) spatially interacts with the CD163 hemoglobin uptake pathway and is a target of inflammatory macrophage activation. J Leukoc Biol.

[37] Delaby C, Pilard N, Puy H, Canonne-Hergaux F (2008). Sequential regulation of ferroportin expression after erythrophagocytosis in murine macrophages: early mRNA induction by haem, followed by iron-dependent protein expression. Biochem J..

[38] Orr JS, Kennedy A, Anderson-Baucum EK, Webb CD, Fordahl SC, Erikson KM (2014). Obesity alters adipose tissue macrophage iron content and tissue iron distribution. Diabetes.

[39] Ahima RS, Flier JS (2000). Adipose tissue as an endocrine organ. Trends Endocrinol Metab.

[40] Matarese G, Moschos S, Mantzoros CS (2005). Leptin in immunology. J Immunol..

[41] Hotamisligil GS, Arner P, Caro JF, Atkinson RL, Spiegelman BM (1995). Increased adipose tissue expression of tumor necrosis factor-alpha in human obesity and insulin resistance. J Clin Invest.

[42] Tack CJ, Stienstra R, Joosten LA, Netea MG (2012). Inflammation links excess fat to insulin resistance: the role of the interleukin-1 family. Immunol Rev.

[43] Bullen JW, Bluher S, Kelesidis T, Mantzoros CS (2007). Regulation of adiponectin and its receptors in response to development of diet-induced obesity in mice. Am J Physiol Endocrinol Metab.

[44] Karlsen A, Retterstol L, Laake P, Paur I, Bohn SK, Sandvik L (2007). Anthocyanins inhibit nuclear factor-kappaB activation in monocytes and reduce plasma concentrations of pro-inflammatory mediators in healthy adults. J Nutr.

[45] Cerletti C, De Curtis A, Bracone F, Digesu C, Morganti AG, Iacoviello L (2017). Dietary anthocyanins and health: data from FLORA and ATHENA EU projects. Br J Clin Pharmacol.

