# Supplementary figures and images for: Purple corn extract induces long-lasting reprogramming and M2 phenotypic switch of adipose tissue macrophages in obese mice
Source: J Transl Med. 2019 Jul 23;17:237. doi: 10.1186/s12967-019-1972-6 (PMC6651915; doi:10.1186/s12967-019-1972-6)

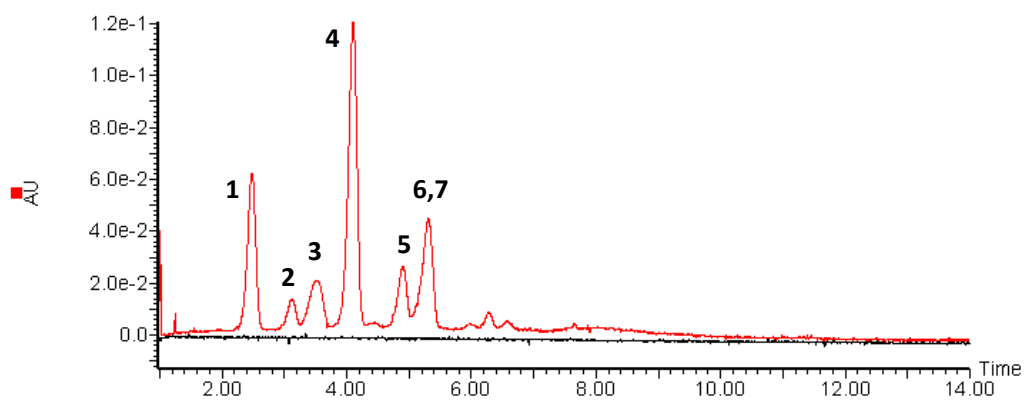

**Figure S1**

A.

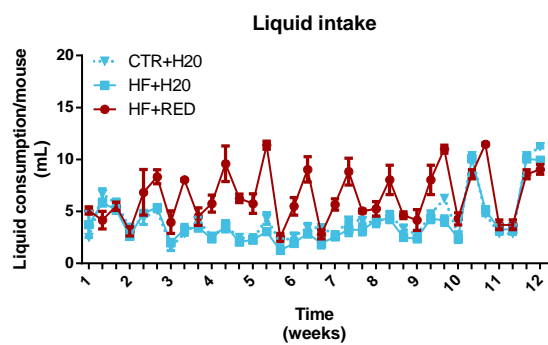

B.

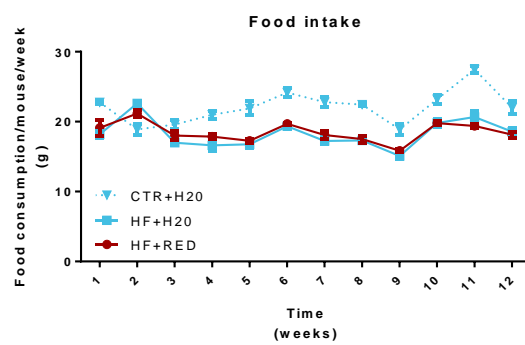

**Figure S2**

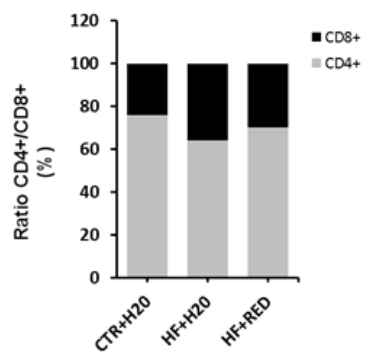

**Figure S3**

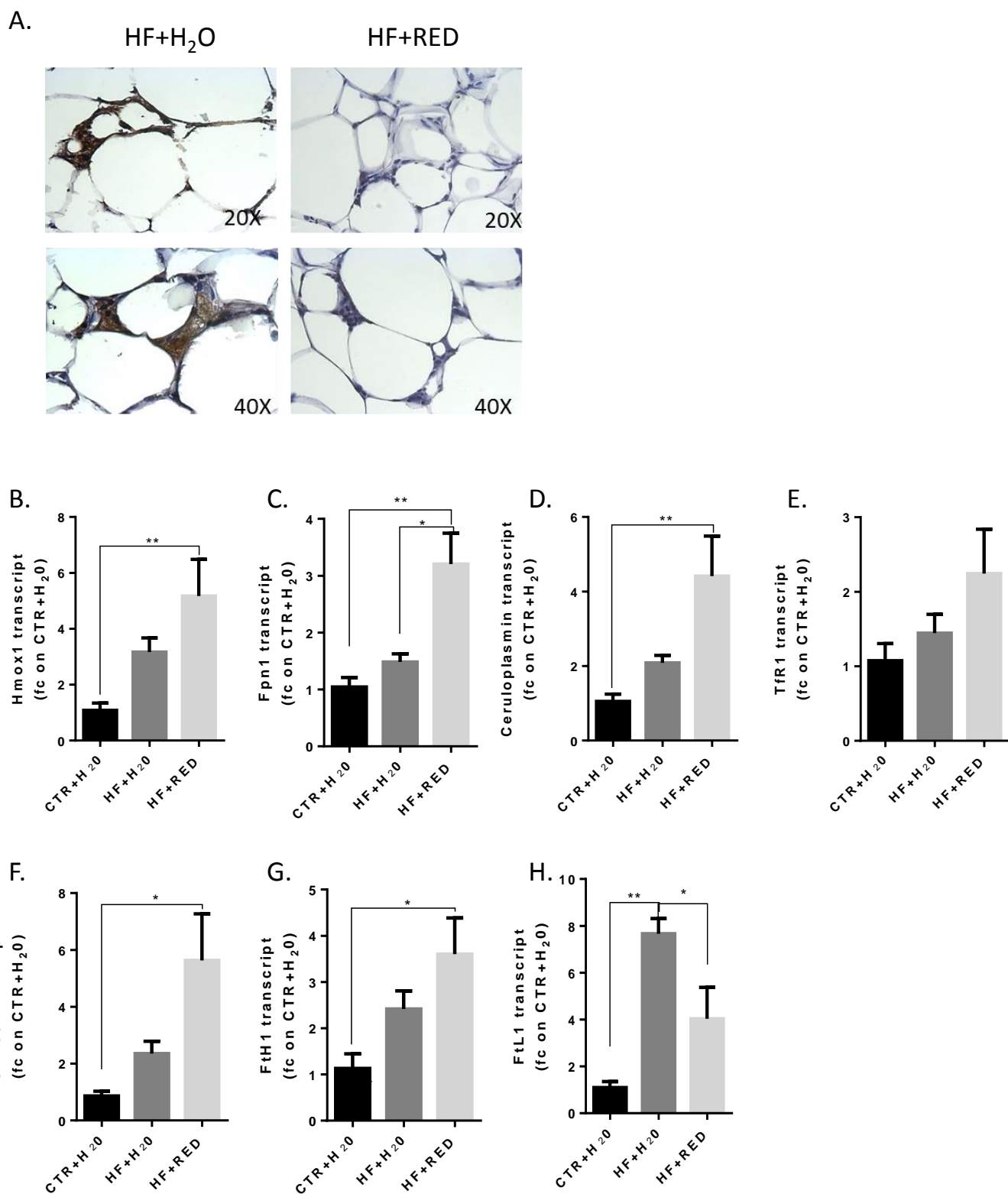

**Figure S4**

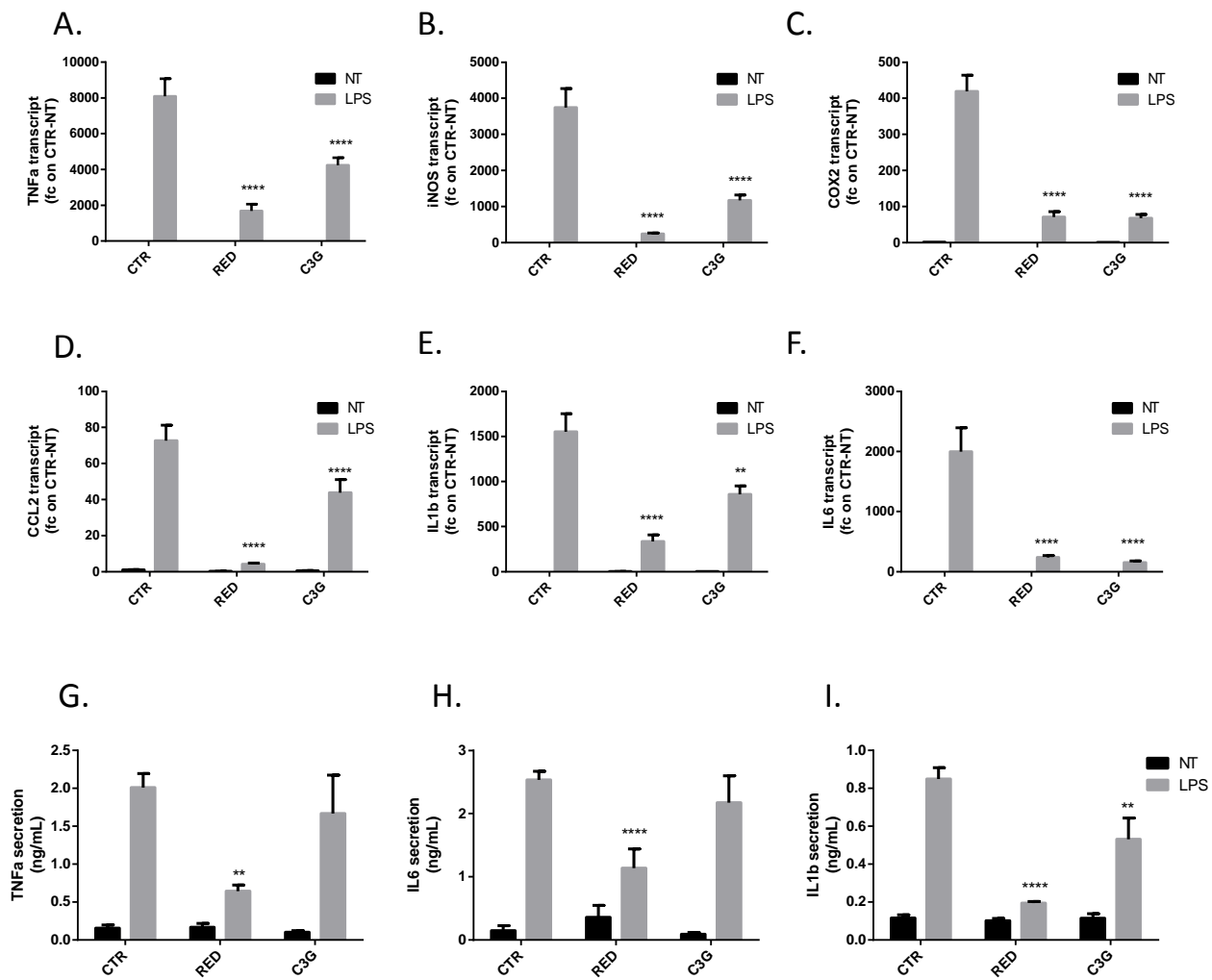

**Figure S5**

Supplement: Supplementary file 1 — Additional file 1: Figure S1. Characterization of the anthocyanin profile and content. Shown in red is a representative HPLC–UV chromatogram from purple corn cob powder at 535 nm detection wavelength. The black line indicates the respective chromatogram of a control sample. Peaks marked with numbers (1 to 7) represent the major anthocyanins whose spectral characteristics, molecular ions and fragments are listed in Additional file 2: Table S1. Figure S2. (A) Liquid intake and (B) food intake of mice fed control or HF diet with or without RED for 12 wk. Mean ± SEM, n = 10. Figure S3. Ratio of CD4+/CD8+ T cells as determined by immunostaining of WAT from mice fed CTR + H2O, HF + H20 or HF + RED. Figure S4. (A) Perls’ Prussian blue staining with DAB intensification of epididymal WAT sections. (B-H) qRT-PCR for the expression of genes involved in iron metabolism. (heme oxygenase-1, Hmox; ferroportin-1, Fpn1; transferrin receptor-1, TfR1; ferritin light, Ftl1 and heavy chains, Fth1). Each transcript was normalized to that in CTR + H2O (n = 6 mice/group). Mean ± SEM. Figure S5. (A-F) qRT-PCR analysis of TNF-α, IL-6, IL-10, COX2, CCL2, IL-1 in PEC cultured with anthocyanin (125 μM) for 24 h and LPS (100 ng/ml) for additional 4 h. (G-I) TNF-α, IL-6 and IL-1β secretion by macrophages primed or not with RED and C3G upon LPS stimulation for 24 h. Mean ± SEM (n = 4 mice/group). [file 12967_2019_1972_MOESM1_ESM.pdf]
