# Supplementary material for: Purple corn extract induces long-lasting reprogramming and M2 phenotypic switch of adipose tissue macrophages in obese mice
Source: J Transl Med. 2019 Jul 23;17:237. doi: 10.1186/s12967-019-1972-6 (PMC6651915; doi:10.1186/s12967-019-1972-6)
Supplement: Supplementary file 2 — Additional file 2. Additional tables. [file 12967_2019_1972_MOESM2_ESM.pdf]

Table S1: Tentative annotation of the major anthocyanins detected in the purple corn cob powder[1]

**Compound**	**R_t**	**UV/Vis λmax**	**LC-ESI MS**	**LC-ESI MS fragments[2]**	**Structure**
	min	*nm*	*(m/z)* $[M+H^+]$		
1	2.48	281/515	449.10	287.13 $(Cy)^+$	Cyanidin-glucoside
2	3.12	280/510	433.11	271.06 $(Pg)^+$	Pelargonidin-glucoside
3	3.53	280/515	463.12	301.07 $(Peo)^+$	Peonidin-glucoside
4	4.10	281/520	535.10	287.13 $(Cy)^+$	Cyanidin-malonylglucoside
5	4.90	280/506	519,11	271.06 $(Pg)^+$	Pelargonidin-malonylglucoside
6	5.27	281/519	549.12	301.07 $(Peo)^+$	Peonidin-malonylglucoside
7	5.32	281/519	621.10	287.05 $(Cy)^+$	Cyanidin-dimalonylglucoside

[1]Peaks 1 to 7 (as indicated in Figure S1**)** were identified based on photo diode array absorbance and mass fragmentation patterns. The purified substances were analysed by HPLC and ESI-MS. Spectral characteristics, molecular ions and fragments of the different compounds are presented.
[2]Fragments: Cyanidin (Cy); Peonidin (Peo); Pelargonidin (Pg).

Gene	Primer sequence	Gene	Primer sequence
Adiponectin	5’-GCACTGGCAAGTTCTACTGCAA-3’ for	CCL2	5’-AGGTCTTCAGCACCTTTGAAT-3’ for
	5’-GTAGGTGAAGAGAACGGCCTTGT-3’ rev		5’-TCACACTGGTCACTCCTACA-3’ rev
Leptin	5’-CCTGTGGCTTTGGTCCTATC-3’ for	F4/80	5’-CGTCAGGTACGGGATGAATATAAG-3’ for
	5’-GATCCTGGTGACAATGGTCTT -3’ rev		5’-ATCTTGGAAGTGGATGGCATAG-3’ rev
Perilipin	5’-GGTGTTACGGATAACGTGGTAG-3’ for	TNFα	5’-GAAAAGCAAGCAGCCAACCA-3’ for
	5’-GATGTCTCGGAATTCGCTCTC-3’ rev		5’-CGGATCATGCTTTCTGTGCTC-3’rev
Resistin	5’-AGAAGGCACAGCAGTCTTG-3’ for	Hmox1	5’-GTCCTGAAGAAGATTGCACAGA -3’ for
	5’-GTCCAGTCTATCCTTGCACAC-3’ rev		5’-GTCGATGTTCGGGAAGGTAAA-3’ rev
Rbp4	5’-AGACAGCTACTCCTTTGTGTTT-3’ for	Fpn1	5’-CGGTCTTTGGTCCTTTGATTTG -3’ for
	5’-CAATCCATCTGTACTGCCTCTC-3’ rev		5’-GCAGAAGGTCAAGAAGGTAGTT-3’ rev
TGFβ	5’-AGAGCGTCTATCACCCATCT-3’ for	CD163	5’-AGTCATCTGCACTGGGAAAG-3’ for
	5’-CTCATCCACCACCATGTCTTC-3’ rev		5’-GACATGAACTCGGAGCAGATAA-3’ rev
Arg1	5’-AGCAGAGGAGGTGAAGAGTA-3’ for	TfR1	5’-TCCTGTCGCCCTATGTATCT-3’ for
	5’-GTAGTCAGTCCCTGGCTTATG-3’ rev		5’-CGAAGCTTCAAGTTCTCCACTA-3’ rev
IL10	5’-GAGGCGCTGTCATCGATTT-3’ for	Cerulopl.	5’-AGAGAGACACTGCAAACCTATTC-3’for
	5’-CACCTTGGTCTTGGAGCTTATT-3’ rev		5’-CCTGTGTAGTGATCCGTTGTAAG-3’ rev
Cox2	5’-CATCCCAGGCCGACTAAAT-3’ for	FtL1	5’-GCCATGGAGAAGAACCTGAA-3’ for
	5’-TGGGCATAAAGCTATGGTTAGA-3’ rev		5’-TTTCCAGGAAGTCACAGAGATG-3’ rev
IL6	5’-CCAGAGTCCTTCAGAGAGATACA-3’ for	FtH1	5’-ACTGCACAAACTGGCTACTG-3’ for
	5’-CCTTCTGTGACTCCAGCTTATC-3’ rev		5’-CGTGGTCACCCAGTTCTTTAAT-3’ rev
IL1b	5’-GGTGTGTGACGTTCCCATTA-3’ for	Fizz1	5’-TGCCAATCCAGCTAACTATCC-3’ for
	5’-ATTGAGGTGGAGAGCTTTCAG-3’ rev		5’-CACACCCAGTAGCAGTCATC-3’ rev

Table S2. Primer sequences utilized for qPCR evaluation of gene expression.

Table S3. Body weight gain, energy intake and feed efficiency[1]

	CTR+H_2O	HF+H_2O	HF+RED
Body weight gain (g)	13.7±0.5	23.2±0.7****	25.3±1.1****
Energy intake (kcal/week)	85.0±1.4	86.4±1.5	87.4±1.1
Feed efficiency[2]	1.3±0.1	2.2±0.1****	2.4±0.2****

[1]Values are mean ± SEM. [2]Body weight gain (g) / kcal consumed. ****p<0.0001 vs CTR+H_2O.

Table S4. Non-esterified fatty acids (NEFA) in plasma of mice under dietary treatments

	Miristic (C14)	Palmitoleic (C16:1)	Palmitic (C16:0)	Linoleic (C18:2)	Oleic (C18:1)	Vaccenic (C18:1)	Stearic (C18:0)	Total NEFA
CTR+H_2O	4.73±1.26	39.46±12.34	33.04±7.29	18.10±2.59	44.03±8.04	10.41±3.00	13.68±3.53	163.46±38.05
HF+H_2O	5.45±0.28	3.41±0.22*	59.03±6.64	42.71±6.40	86.94±21.69**	10.42±0.80	42.84±8.07	250.80±39.20
HF+RED	3.78±0.66	2.67±0.47*	41.32±4.18	32.43±4.67	72.39±11.30	8.27±1.44	29.72±6.09	190.59±26.58

Concentrations are expressed as ng/mL. Results are expressed as mean ±SEM (n=5-6). *, $p<0.05$, **, $p<0.01$ vs CTR+H_2O.
